# COVID-related constrictive pericarditis requiring pericardiectomy: a case report

**DOI:** 10.1186/s13019-024-02950-1

**Published:** 2024-07-13

**Authors:** Rachel Boyles, Joseph Lu, Joseph Yoo, Louis Samuels

**Affiliations:** 1 Department of Surgery, Division of Cardiac Surgery, Jefferson-Einstein Medical Center, Philadelphia, PA USA; 2 Jefferson-Einstein Medical Center, 5501 Old York Road, Klein Building—Suite 401, Philadelphia, PA 19141 USA

**Keywords:** COVID-19, Constrictive pericarditis, Pericardiectomy

## Abstract

**Background:**

The COVID-19 pandemic was primarily considered a respiratory malady in the early phases of the outbreak. However, as more patients suffer from this illness, a myriad of symptoms emerge in organ systems separate from the lungs. Among those patients with cardiac involvement, myocarditis, pericarditis, myocardial infarction, and arrhythmia were among the most common manifestations. Pericarditis with pericardial effusion requiring medical or interventional treatments has been previously reported in the acute setting. Notably, chronic pericarditis with pericardial thickening resulting in constriction requiring sternotomy and pericardiectomy has not been published to date.

**Case presentation:**

A patient with COVID-19-associated constrictive pericarditis three years after viral infection requiring pericardiectomy was reported. The COVID-19 infection originally manifested as anosmia and ageusia. Subsequently, the patient developed dyspnea, fatigue, right-sided chest pressure, bilateral leg edema, and abdominal fullness. Following recurrent right pleural effusions and a negative autoimmune work-up, the patient was referred for cardiothoracic surgery for pericardiectomy when radiographic imaging and hemodynamic assessment were consistent with constrictive pericarditis. Upon median sternotomy, the patient’s pericardium was measured to be 8 mm thick. Descriptions of the clinical, diagnostic, and therapeutic features are provided. Within the first week after the operation, the patient’s dyspnea resolved; one month later, leg edema and abdominal bloating were relieved.

**Conclusions:**

Although an association between COVID-19 and cardiac complications has been established, this case adds another element of virus severity and chronic manifestations. The need for sternotomy and pericardiectomy to treat COVID-19-related constrictive pericarditis is believed to be the first reported diagnosis.

## Introduction

The COVID-19 pandemic primarily began as a respiratory illness, killing nearly 7 million people worldwide since its outbreak [1]. As the infection spread and more patients were afflicted, the extrapulmonary manifestations grew exponentially. The cardiovascular system was not spared; rather, it was a reaction related directly to the virus and indirectly to the body’s immune response. Although the focus of attention was primarily on respiratory disease in early experience, cardiovascular effects were also encountered in some of the first reports from China. In a manuscript describing forty-one patients at the outset of the pandemic, Huang et al. reported a 12% incidence of acute cardiac injury [2]. As the pandemic spread, cardiac manifestations became more apparent with a spectrum of pathologies, including myocarditis, pericarditis, arrhythmia, and myocardial infarction—these conditions occasionally lead to heart failure and death. In 2021, for example, Halushka and colleagues reported on 277 postmortem examinations of COVID-19 patients and found myocarditis in 7.2% of the autopsies [3]. Although relatively uncommon, myocarditis is sometimes associated with pericarditis, a condition appropriately termed myopericarditis or perimyocarditis. The clinical presentations of this combined state varied from subclinical to cardiogenic shock. Depending on the severity of the condition, therapies included medications (e.g., steroids, nonsteroidal-anti-inflammatory drugs), antiviral agents, and immune-modulating chemotherapeutics. More serious cases required interventional/surgical treatments (e.g., pericardiocentesis, pericardial window) and mechanical cardiac support technologies (e.g., IABP, VAD, ECMO). For instance, Walker and others treated a patient with COVID-19 who was infected at 30 years of age with pericardial tamponade utilizing a surgical subxiphoid pericardial window [4]. Samuels and colleagues managed a 58-year-old man with COVID-19 who presented with cardiogenic shock with a percutaneous ventricular assistive device (VAD) followed by extracorporeal membrane oxygenation (ECMO) [5]. These case reports added to the spectrum of cardiac disease processes associated with COVID-19 and the treatments necessary to manage them in the acute phase. The consequences of the infection beyond the initial weeks and months of disease onset was unknown at the time.

As the world community has experienced years of disease, the lingering effects of COVID-19—the so-called ‘long COVID-19’—have begun to appear. Persistent symptoms remained in subsets of patients—some were minimally debilitating, while others were crippling. Some of the acute cardiovascular consequences of the infection are chronic problems, such as COVID-19-related cardiomyopathy. The purpose of this report is to describe another cardiac pathology associated with COVID-19: constrictive pericarditis. This additional chronic entity, constrictive pericarditis, can now be added to the growing list of cardiac COVID-19 cases. The clinical presentation and diagnosis of this condition, along with the findings and treatment with pericardiectomy, are described in this case report. We believe that this is the first report of this condition—COVID-19-associated constrictive pericarditis requiring pericardiectomy.

## Case

**Image 1:**
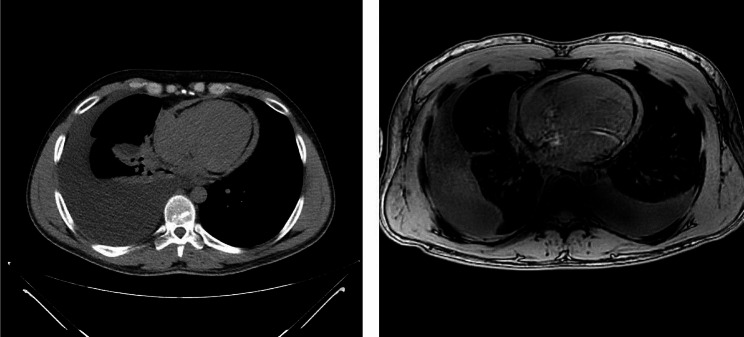
**A** and **B** CT scan and MRI demonstrating pericardial thickening.

A 37-year-old man with symptoms of anosmia and ageusia contracted COVID-19 in October 2020. Over the next two years, he began to complain of fatigue, dyspnea, and right-sided chest pressure. Chest radiography demonstrated moderate right pleural effusion. He was referred to the pulmonary clinic in November 2022, where thoracentesis was recommended. The next week, right thoracentesis yielded 2250 mL of serosanguinous fluid. A month later, in December 2022, a chest CT scan demonstrated the recurrence of moderate to large right pleural effusion and a small pericardial effusion with high density, suggestive of hemopericardium or exudative effusion from prior pericarditis (Image 1a). He was referred for thoracic surgery. Prior to the performance of a surgical drainage procedure, transthoracic echocardiography demonstrated a normal left ventricular ejection fraction (LVEF) of 55%, normal RV size and function, and no valvulopathy. However, the mitral annular velocity was consistent with the annulus reversal, suggesting constrictive physiology. In addition, there was a small pericardial effusion localized posteriorly, and the inferior vena cava (IVC) failed to collapse with forced inspiration. In January 2023, Video-Assisted thoracoscopic surgery (VATS) was performed, yielding 2700 mLs of serous pleural fluid from the right chest. The operative findings included mild thickening and inflammation of the parietal pleura. Pleural biopsies revealed adipose tissue with lymphoid aggregates. The pleural fluid showed rare lymphocytes and rare mesothelial cells; there was no growth from the cultures, and cytology was negative. Although she initially improved from a respiratory standpoint, the patient began to experience dyspnea again along with abdominal bloating and leg swelling several months later. A chest radiograph taken on August 2023 demonstrated recurrence of the right pleural effusion. Additional studies were undertaken.

Abdominal MRI was used to evaluate the patient’s gastrointestinal symptoms, as was blood chemistry, which revealed elevated liver function tests (Image 1b). Images of the lower chest showed bilateral pleural effusions, and the pericardium demonstrated a progressive thin enhancement suggestive of underlying pericarditis. There was no change in the proteinaceous or hemorrhagic pericardial effusion observed on the prior CT scan. Finally, there was evidence of mild to moderate ascites. The patient was referred for interventional radiology, where a liver biopsy was performed revealing central venous sinusoidal dilatation along with nodular regenerative hyperplasia. Focal portal and periportal fibrosis with periportal metaplastic hepatocytes was also observed. These hepatic findings were consistent with cardiac origin. As such, the patient was referred to a cardiologist. In October 2023, right and left heart catheterization were performed with the following findings:

1. Diastolic equalization of pressure was achieved, with elevated left ventricular end-diastolic pressure (LVEDP) and right ventricular end-diastolic pressure (RVEDP) and pulmonary capillary wedge pressure.
2. A steep X and Y descent, a square root sign, and a LVEDP greater than 1/3 of the total RV systolic pressure are consistent with impaired diastolic filling and are often accompanied by pericardial constriction.
3. Vagal events during the procedure requiring IV fluids, atropine and neonephrine.
4. Normal epicardial coronary arteries with very slow TIMI II flow, suggestive of myocardial/microvascular dysfunction.

In view of these hemodynamic findings, along with the radiographic imaging and clinical features, a diagnosis of constrictive pericarditis was made, and the patient was referred for cardiac surgery for pericardiectomy.

**Image 2:**
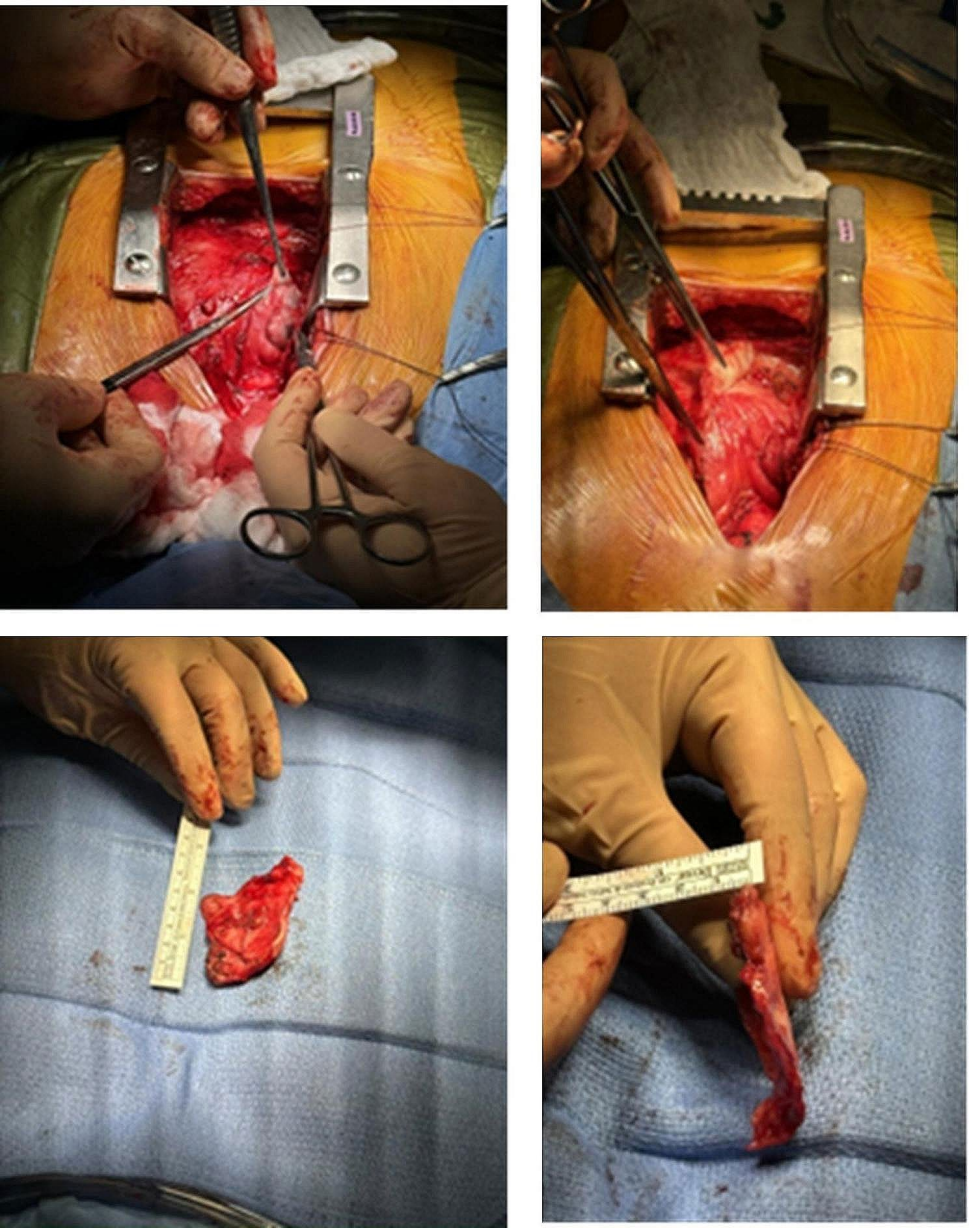
**A** Dissection of the pericardium from the right atrium and right ventricle. **B** Portion of pericardium.

The patient was taken to the operating room on October 13, 2023—three years after his initial COVID-19 infection—at which point he underwent median sternotomy and pericardiectomy. Cardiopulmonary standby was available but not utilized. The operative findings included a thickened pericardium (8 mm) that was densely adherent to the epicardium (Image 2a and 2b). Several hours of dissection were necessary to safely resect the pericardial tissue with particular care to release the superior vena cava (SVC) and inferior vena cava (IVC) as well as the heart while avoiding injury to the phrenic nerves. The right atrial pressure prior to pericardial resection was 21 mmHg and 9 mmHg at the end of the case. In addition, the right pleural space was opened, and 500 mL of serous fluid was evacuated. A mediastinal lymph node was also sampled. Pathology of the pericardium revealed fibrous tissue consistent with chronic inflammation; the mediastinal lymph node demonstrated sinus histocytes and interfollicular polyclonal plasmacytosis. The patient’s postoperative course was uncomplicated, and he was discharged on a regimen of colchicine. His shortness of breath resolved within the first week, and outpatient follow-up one month later was notable for the resolution of all the symptoms, including abdominal bloating and leg edema.

## Discussion

With the spread of the COVID-19 pandemic in two waves across diverse populations throughout the world, the long-term impact of this viral infection on the heart is yet to be fully understood. By examining the features of previous cases as well as newly reported cases, clinicians may better understand the various manifestations of COVID-19 infection, as it pertains to myopericarditis. This case report adds to the short list of similar cardiac complications experienced by others. Two publications, for example, reported on a review of COVID-19-related cardiac disease in general and commented on some cases of myopericarditis in men under 50 years of age [6, 7]. Another publication highlighted the case of an 80-year-old man with constrictive pericarditis who presented with shortness of breath, peripheral edema, weight loss, and skin rash. However, this case is the first known patient to require a pericardiectomy. COVID-19-related constrictive pericarditis was diagnosed by the need for a VATS procedure in which the pleural effusion was drained and a partial anterior pericardiectomy was performed. Pathology of the specimen revealed an autoimmune or immunoglobulin etiology [8]. These manuscripts postulate an immunologic process as the mechanism by which the inflammatory pathways of the host result in clinical and histologic findings within the pericardium and elsewhere. Therefore, treatments were a combination of medical and surgical interventions.

The literature suggests that the pathophysiology of COVID-19 involving the pericardium lies within its ability to stimulate innate immune system pathways, such as complement, TLR4, and inflammasome pathways. In this milieu, there are increases in IL-1β and IL-18, which have been shown to be enhanced in men. This pathway may be responsible for some of the dilated cardiomyopathies caused by viruses and may provide an explanation for why men under 50 years of age develop myopericarditis in the setting of COVID-19 [7]. Studies have also investigated the relationship between the mRNA vaccine developed for the COVID-19 virus and the incidence of myocarditis and pericarditis. One such study reviewed 29 published cases of mRNA vaccine-induced myopericarditis. The cases reviewed in this manuscript described chest pain occurring within 1–5 days of the second dose [9]. Similarly, the patients in this review exhibited a common trend toward younger males. This study speculates on the pathogenesis attributed to the increased antibody reactivity found in a portion of younger individuals, which may enhance the response parallel to the multisystem inflammatory syndrome seen in children (MIS-C), a reaction leading to a more severe response to the virus.

In summary, the case of COVID-19-related pericarditis requiring pericardiectomy three years after the initial infection represents another pathology to be added to the growing list of COVID-related cardiac diseases. Clinicians need to be vigilant in suspecting and treating patients who present with acute signs and/or symptoms of pericarditis to avoid long-term chronic consequences such as constrictive pericarditis requiring pericardiectomy.

## Data Availability

No datasets were generated or analysed during the current study.

## Abbreviations

VAD: ventricular assistive device
ECMO: extracorporeal membrane oxygenation
VATS: Video-Assisted thoracoscopic surgery
LVEDP: left ventricular end-diastolic pressure
RVEDP: right ventricular end-diastolic pressure
NAFLD: Nonalcoholic Fatty Liver Disease
